# Restoration of intestinal continuity after stoma formation for Crohn’s disease in the era of biological therapy: A retrospective cohort study

**DOI:** 10.1007/s00508-019-01586-9

**Published:** 2020-01-08

**Authors:** Catharina Müller, Michael Bergmann, Anton Stift, Stanislaus Argeny, Doug Speake, Lukas Unger, Stefan Riss

**Affiliations:** 1 Division of General Surgery, Department of Surgery, Medical University of Vienna, Währinger Gürtel 18–20, 1090 Vienna, Austria; 2 Department of Surgery, Western General Hospital, Crewe Road South, EH4 2XU Edinburgh, UK

**Keywords:** Perianal disease, Ostomy, Fistula, Surgery, Biologic

## Abstract

**Background:**

The rate of restoration of intestinal continuity after colonic resection and stoma creation in patients with Crohn’s disease has not been well-documented in the era of biologics. Thus, the incidence of restoration of intestinal continuity since the introduction of biological drugs was assessed.

**Methods:**

Consecutive patients (*n* = 43) who underwent colonic resection with ileostomy or colostomy formation for Crohn’s disease at a single tertiary referral center between 2002 and 2014 were identified. Data from individual chart review were analyzed retrospectively. Patients were personally contacted for follow-up.

**Results:**

Of the 43 patients 8 (18.4%) had a proctectomy leaving 35 patients (81.4%) with the rectum preserved. Of the 30 patients qualifying for final analysis restoration of bowel continuity was finally achieved in 10 patients (33.3%). Permanent stoma rates were comparable in the group of patients with and without biological therapy after surgery (64.3% vs. 60%). The median follow-up period was 7 years (range 3–15 years). Of the patients 20 suffered from perianal disease involvement (66.7%), which was associated with a higher rate of permanent stoma (*n* = 16/20, 80%) in contrast to patients without perianal disease (*n* = 4/10, 40%, *p* = 0.045).

**Conclusion:**

The overall incidence of stoma formation was low for patients with Crohn’s disease; however, once a stoma is created the chance of ending up with a permanent stoma is high even in the era of biologics. Despite the use of new therapeutic agents perianal disease increases the risk of a permanent stoma.

## Introduction

Patients suffering from Crohn’s disease (CD) have a high risk of requiring surgery with up to one third having bowel resection due to CD-related complications within 5 years of diagnosis [1]. Ileocolic resection is the most common procedure conducted in CD but colectomy may be needed in selected patients. Particularly in complicated CD with perforation or severe rectal or perianal involvement the creation of an ileostomy or colostomy may be necessary. Notably, in those patients with ongoing perianal sepsis and rectal stump inflammation restoration of intestinal continuity may be delayed or not achievable. In the prebiologic era the probability of a definitive stoma for patients with CD could reach 14% after 20 years from disease onset [2]. For isolated colonic CD, rates of permanent stoma are even higher with up to 31.4% after total and 34.7% after segmental colectomy [3]. Patients with CD are often young at the time of surgery and stoma formation may significantly restrict the quality of life [4].

Few studies have reported about the overall probability of subsequent restoration of intestinal continuity after stoma creation in patients with CD and outcome varies in the literature. While stoma reversal rates for loop ileostomy have uniformly been found to be about 90% [5, 6], the rate of successful intestinal reconstruction after colectomy and terminal ileostomy varies widely (8–100%) [5, 7, 8].

The treatment of CD has changed significantly over the last 25 years. With the introduction of biological drugs, such as tumor necrosis factor alpha (TNF-alpha) inhibitors, a reduction of intestinal resections due to CD has been noted [9]. These findings are most significant for the first year after biological therapy initiation [10, 11]; however benefit beyond this period regarding the rate of surgery in the long-term has been recently questioned [1, 12]. Moreover, it remains unclear whether the use of biological drugs subsequently impacts the probability of intestinal reconstruction after stoma formation in CD [9, 13–15]. It was hypothesized that the administration of novel immunosuppressive medication improves disease control postoperatively and increases the chance of restoration of bowel continuity after stoma formation. The study aimed to evaluate the clinical course and likelihood of intestinal reconstruction after stoma formation in patients with CD in the era of biological drugs.

## Method

A total of 342 consecutive patients were identified, who underwent intestinal resection due to CD at a single tertiary referral center, the Division of General Surgery, Department of Surgery, Medical University of Vienna, between 2002 and 2014. In 43/342 (12.6%) patients a colonic resection with ileostomy or colostomy formation was conducted (ileostomy *n* = 28, 65%, colostomy: *n* = 15, 35%). Of the patients 8 (*n* = 8/43, 20.4%) underwent proctectomy or proctocolectomy not qualifying for subsequent direct restoration of bowel integrity and 5 patients had to be excluded because they were lost for follow-up. Thus, 30 patients were available for final analysis. The median follow-up period was 7 years (range 3–15 years). All operations were performed by a single colorectal surgical team specialized in the management of CD. Each patient was discussed in a multidisciplinary team meeting for inflammatory bowel disease prior surgery. Data were retrieved from the institutional database and individual chart reviews. All patients qualifying for final analysis were personally contacted by phone and a structured interview was performed for completion of follow-up. The study was approved by the institutional ethics committee (ECS 1498/2018).

Baseline characteristics and surgical data consisting of type of operation (subtotal colectomy or colostomy), type of stoma (colostomy, terminal ileostomy, protective ileostomy), complexity of surgery (simple involving only one resection and complex with the need of more than one resection and/or additional strictureplasty), surgical approach (open, laparoscopic, conversion as defined previously [16]), urgency of operation (elective versus emergency operation) and indications were recorded. Perianal disease was defined as the presence of a perianal fistula and/or abscess formation.

### Statistical considerations

The IBM Statistical Package for the Social Sciences (SPSS) version 24 for Mac (SPSS, Chicago, IL, USA) was used for statistical analysis. Descriptive data were represented as frequencies and percentages for categorical and as median and range for continuous variables as applicable. Due to the sample size 2‑sided Fisher’s exact test was used for calculation of differences for categorical parameters and Student’s t‑test for continuous variables. A two-sided *p*-value of <0.05 was considered to denote statistical significance.

## Results

### Baseline characteristics

Baseline characteristics of the finally included patients (*n* = 30) are outlined in Table 1.

**Table 1:** Patient baseline characteristics

**Patient characteristics**	
*Gender*	
Male	15 (50%)
Female	15 (50%)
*Age at time of surgery*	
	36.9 years (range 15–61 years)
*Body mass index (BMI)*	
	20.5 kg/m^2^ (range 14.5–28.2 kg/m^2^)
*Smoking*	
Yes	14 (46.7%)
No	16 (53.3%)
*Biological treatment preoperatively*	
Yes	13 (43.3%)
No	11 (36.7%)
*Biological treatment postoperatively*	
Yes	10 (33.3%)
No	14 (46.7%)
*Glucocorticoids preoperatively*	
Systemic	11 (36.7%)
Locally	1 (3.3%)
Combined	2 (6.7%)
*Glucocorticoids postoperatively*	
Systemic	9 (30%)
Locally	1 (3.3%)
Combined	1 (3.3%)
*Azathioprine preoperatively*	
Yes	14 (46.7%)
No	11 (36.7%)
*Azathioprine postoperatively*	
Yes	14 (46.7%)
No	8 (26.7%)
**Disease characteristics**	
*Perianal involvement*	
Perianal disease	20 (66.7%)
No perianal disease	10 (33.3%)
*Penetrating disease*	
Yes	16 (46.7%)
No	14 (63.3%)
**Surgical characteristics**	
*Type of initial stoma*	
Terminal ileostomy	16 (53.3%)
Protecting ileostomy	2 (6.7%)
Colostomy	12 (40%)
*Operation time*	
	185 min (90–495)
*Urgency of operation*	
Acute	13 (43.3%)
Elective	17 (56.7%)
*Complexity*	
Simple	22 (73.3%)
Complex	8 (26.7%)
*Approach*	
Open	17 (56.7%)
Laparoscopic	7 (23.3%)
Conversion	6 (20%)

Terminal ileostomy was fashioned for 16 patients (53.3%), 12 patients had a colostomy (40%) performed and 2 patients had a loop ileostomy (6.7%). The indications for stoma formation were fistula as such for 11 patients (36.7%) and incontinence for 1 patient (3.4%), 13 patients (43.3%) received a stoma in the context of acute surgery. For the rest of the patients (*n* = 5, 16.7%) either intraoperative technical factors or patient factors made stoma creation necessary (Table 2).

**Table 2:** Indications for stoma formation in detail

Elective *n* = 17 (56.7%)		Acute *n* = 13 (43.3%)	
Fistula	*n* = 11 (36.7%)	Anastomotic leak	*n* = 6 (20%)
Stenosis	*n* = 2 (6.8%)	Bowel perforation	*n* = 5 (16.7%)
Therapy refractory disease	*n* = 2 (6.8%)	Therapy refractory disease	*n* = 1 (3.4%)
Abscess	*n* = 1 (3.4%)	Bleeding	*n* = 1 (3.4%)
Incontinence	*n* = 1 (3.4%)	–	–

### Fate of the remaining rectum and restoration of bowel continuity

**Fig. 1:**
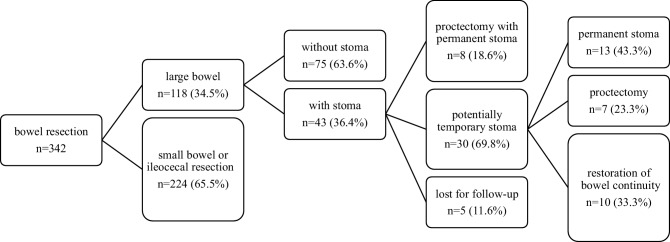
Flowchart of stoma formation after bowel resection and restoration of bowel continuity

Of the 30 patients analyzed 13 (43.3%) had to undergo repeated intestinal resection and of those, 7 patients (53.8%, overall 23.3%) required resection of the remaining rectum. Only 10 (33.3%) patients underwent subsequent stoma closure during the follow-up period. In detail, all patients (*n* = 2) with a protecting ileostomy at the index operation underwent later stoma closure. Of those patients with a terminal ileostomy 11 (*n* = 11/16, 68.8%) were left with a permanent stoma (*n* = 11) while 75% of patients after colostomy (*n* = 9/12) did not qualify for subsequent stoma closure. In total, 20/30 patients (66.7%) were left with a permanent stoma (Fig. 1).

### Effect of biological therapy on stoma closure rate

The use of immunosuppressive treatment is listed in Tables 1 and 3.

**Table 3:** Stoma reversal and biological treatment. Rate of restoration of bowel continuity according to preoperative and postoperative biological treatment

Preoperative/postoperative biologicals	Restoration of intestinal continuity	Permanent stoma
No/yes	*n* =2 (33.3%)	*n* =4 (66.7%)
Yes/no	*n* =1 (25%)	*n* =3 (75%)
Yes/yes	*n* =2 (28.6%)	*n* =5 (71.4%)
No/no	*n* =2 (40%)	*n* =3 (60%)

Of the patients 14 (46.7%) received biologics after surgery, 4 patients (28.6%) were treated with infliximab, 6 patients (42.9%) had adalimumab and 3 patients (21.4%) received ustekinumab. Rates of permanent stoma were similar for patients with or without biological therapy. In 60% of the patients (*n* = 6) without adjuvant biological therapy following colonic resection the stoma could not be reversed, which was comparable to 64.3% of patients (*n* = 9) who received biological treatment, 3 patients (21.4%) in the biological treatment group underwent proctectomy during the follow-up period, while only 1 patient (10%) without biologics had a proctectomy (*p* = 0.615).

Patients having azathioprine tended to have a higher rate of permanent stoma (*n* = 10/14, 71.4%) compared to patients (*n* = 4/8, 50%) without azathioprine postoperatively, without reaching statistically significance (*p* = 0.638).

Biological treatment prior to surgery did not significantly reduce the risk of a permanent stoma (*p* = 0.659, 76.9% vs. 63.6%). Besides medical treatment other factors, such as age (*p* = 0.291), BMI (*p* = 0.980) and smoking (*p* = 0.709) were not found to influence the overall rate of stoma reversal.

### Influence of perianal disease on intestinal reconstruction rate

**Fig. 2:**
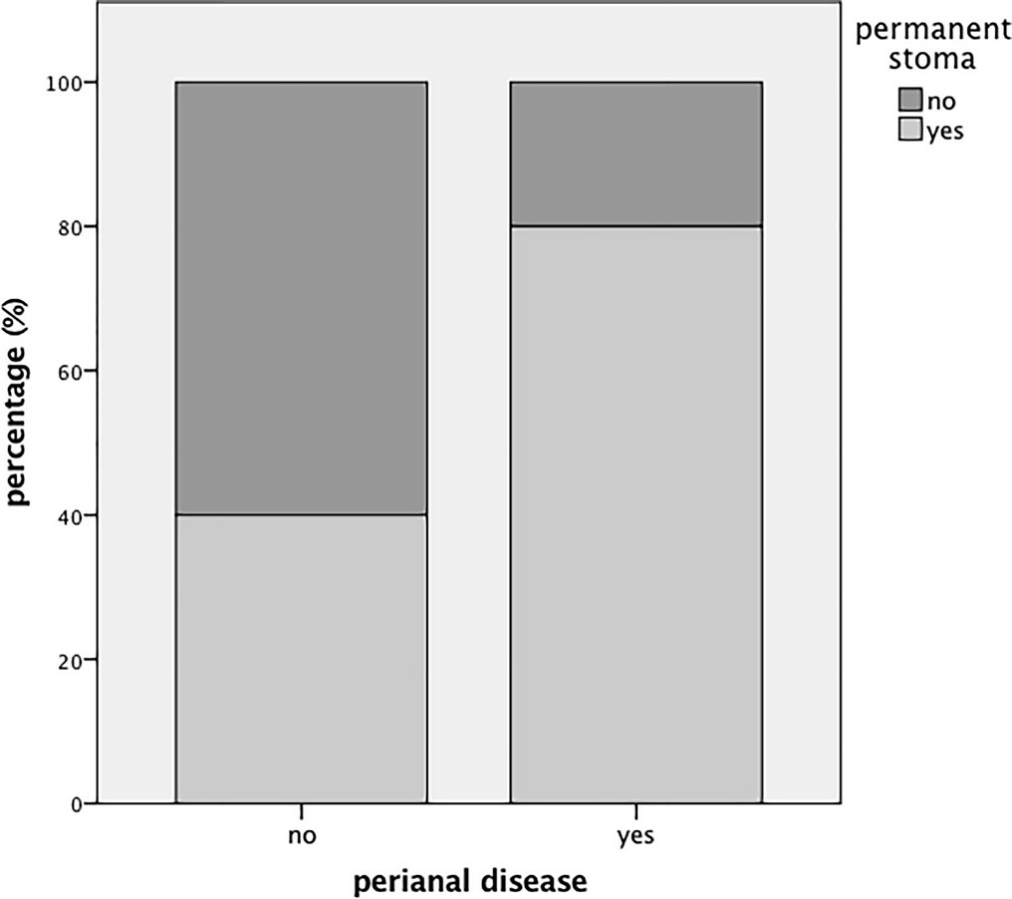
Chance of permanent stoma in the presence of perianal disease involvement

In the present series 20 patients (66.7%) suffered from perianal disease involvement, which was associated with a significantly increased rate of permanent stoma creation (*n* = 16/20, 80%) in contrast to patients without perianal disease (*n* = 4/10, 40%, *p* = 0.045, Fig. 2). In the group of patients having a stoma performed with concomitant perianal disease six patients (*n* = 6/20, 30%) underwent further proctectomy. In contrast only one patient lacking perianal disease involvement finally had to undergo proctectomy (*n* = 1/10, 10%). Biological drugs were administered in 73.3% of patients with perianal disease, while only 33.3% of patients lacking perianal involvement received biologics (*p* = 0.092).

## Discussion

In the present study it could be demonstrated that CD patients, who required stoma formation due to severe CD, still had a high risk of permanent stoma despite the use of new biological treatment. This is an interesting clinical finding as it could be speculated that patients may have a higher chance of stoma reversal as medical treatment improves. Especially, patients with perianal CD still have a significant chance to end up with a definite stoma. There are various reasons for patients with CD to have a stoma created. In this cohort it was mainly due to disease-related complications. Especially patients with a fistula accounted for nearly half of the complications (45.8%), six patients (20%) had a stoma performed as a result of a complicated postoperative course after bowel resection with primary anastomosis.

The overall risk of permanent stoma formation with changing treatment strategies and new therapeutic options for patients with CD over time is under debate. Cosnes et al. found no difference in the rate of definitive stoma formation in patients having bowel resection due to CD over time (1987–2002) even after the routine use of biologics [15]; however, Ramadas et al. found a reduced number of patients ending up with a stoma 5 years after initial diagnosis associated with the change in medical treatment and the widespread use of immunosuppressive agents in their population-based study from Cardiff over the same time period. Notably they focused on era effects and early postoperative thiopurine administration and described a rate of only 16% of patients receiving biologics [9].

Coscia et al. compared 51 patients with Crohn’s colitis in the prebiologic era with 182 patients in the era of biologics who underwent bowel resection. They found significantly lower rates of patients with permanent ileostomy (19.1%) in the era of biological drugs as compared to 60.8% in the prebiologic era [13]. Interestingly, differences were less significant when comparing patients with and without administration of biological agents. This may indicate that not only the improvement of medical treatment but also other factors potentially influence stoma reversal rate over time, such as differences in disease manifestation, severity of disease, smoking habits or overall surgical strategy and timing of surgery. As an example, Coscia et al. [13] found higher rates of initial proctocolectomy in the prebiologic era than in the era of biological drugs (33.3% vs. 5%) indicating less extensive surgical treatment over time. In contrast to previous studies this focused on just one era—the era of biologics and found that permanent stoma rates were comparable between the group of patients receiving biological treatment after surgery (64.3%) and those without biological treatment (60%).

Data based on the largest US inpatient database including 335,239 CD-related surgeries showed an increasing rate of overall stoma creation despite the rising use of biologics [14]. The authors speculated that patients may be more ill at the time of the operation as they present later for surgery, resulting in creation of more stomas. They do not further differentiate between terminal and protecting ileostomies. A rise of the latter could indicate a higher rate of ileorectal anastomosis over time with eventually more stoma closure in the future. In the prebiologic era Yamamoto and Keighley described 50% of colectomies performed in a two-step procedure with the need for terminal ileostomy in the primary operation [8]. The present study found approximately 30% of patients with stoma creation at the time of large bowel resection.

Perianal disease involvement may influence the stoma reversal rate. Perianal disease is common and affects about one third of patients with CD. If surgery for perianal disease is needed the quality of life has been shown to be restricted [17]. Furthermore, patients with perianal CD have a 30–50% risk of permanent stoma [18, 19] and approximately 20% undergo further proctectomy. Patients with perianal fistula especially benefit from novel medical strategies [20]. Whether biologics reduce the stoma rate for patients with perianal disease is under debate. In this study 67% of patients in the final cohort suffered from perianal involvement. In line with previously published data, only about 20% of patients with perianal disease underwent subsequent stoma reversal, which was in contrast to 60% of patients without an anal fistula. The rate of successful stoma reversal remained low, although the vast majority of this group of patients received biologics after surgery. These findings are in line with a case series of 21 patients that reported no improvement in stoma reversal rates after intended temporary fecal diversion owing to biological therapy [21]. This could be strengthened by a larger study retrospectively observing 138 patients with severe perianal CD and showing no association with biological treatment and increased intestinal reconstruction [22] and also a systemic review evaluating the rate of stoma closure after intended temporary fecal diversion for severe perianal CD did not find a better outcome for patients in the era of biologics [23].

Patients having a proctectomy may suffer from sexual dysfunction due to sexual nerve damage or problems with perianal wound healing due to pelvic dissection. Thus, preserving the rectal stump may not only maintain the possibility of subsequent bowel restoration but also prevent patients from several side effects. Approximately 25% of patients with an initially preserved rectal stump finally underwent proctectomy. These rates were even higher for patients with perianal disease (30%). The rate of proctectomy decrease slightly compared to the findings in the prebiologic era. Harling et al. found a cumulative risk of proctectomy of 50% for CD patients after colectomy and terminal ileostomy after 10 years [7]. Accordingly, Cattan et al. reported 51% of patients needing a proctectomy after colectomy with terminal ileostomy and a preserved rectal stump in the prebiologic era [24]. In the present cohort the proctectomy rate was 23.3% after a median follow-up of 7 years.

The current study has some limitations that need to be addressed. Although, it included a large cohort, the actual number of CD patients with a stoma formation remained low; however, taking into account the small case series in the present literature, we are still convinced of the clinical importance of the results. Data about the immunosuppressive medications were partly recorded retrospectively and might potentially bias the outcome. Noteworthy, all patients’ charts were carefully studied and all patients were contacted personally to minimize any deviations.

## Conclusion

Despite continuous use of biological drugs in CD the chance of ending up with a permanent stoma remains high once a stoma is created, as only about one third of patients undergo subsequent restoration of bowel continuity. Patients with perianal disease are significantly more likely to be left with a permanent stoma irrespective of treatment with biologics.

## Abbreviations

CD: Crohn’s disease
IC: Ileostomy closure
IPAA: Ileopouchanal anastomosis
IRA: Ileorectal anastomosis
SC: Stoma closure
SPSS: Statistical Package for the Social Sciences
TC+I: Total colectomy with terminal ileostomy
